# Micro-Computed Tomography Assessment of Voids and Volume Changes in Bulk-Fill Restoration with Stamp Technique

**DOI:** 10.3390/ma18174027

**Published:** 2025-08-28

**Authors:** Ralitsa Gigova, Krasimir Hristov

**Affiliations:** Department of Pediatric Dentistry, Faculty of Dental Medicine, Medical University of Sofia, 1431 Sofia, Bulgaria; r.bogovska@fdm.mu-sofia.bg

**Keywords:** stamp technique, bulk-fill composite, occlusal restoration, void formation, micro-CT analysis

## Abstract

The stamp technique with bulk-fill composites aims to enhance occlusal surface replication in Class I restorations. Limited research exists on its void formation and volumetric changes. This study measures internal and external voids as well as volumetric changes in occlusal surfaces for both the stamp and conventional bulk-fill techniques. Materials and methods: Twenty-four permanent molars were divided into two groups (n = 12 each): Group 1 (conventional bulk fill) and Group 2 (stamp technique with bulk-fill composite). Standardized Class I cavities were prepared and restored using Tetric EvoCeram^®^ Bulk Fill composite. Micro-CT scanning was performed before and after restoration to quantify internal and external void percentages and volumetric changes. An independent samples *t*-test (α = 0.05) was used to compare void percentages and volumetric changes between groups. Results: The mean internal void percentage was similar between groups (Group 1: 0.38 ± 0.22%; Group 2: 0.39 ± 0.30%; *p* = 0.914), indicating comparable internal adaptation. Group 2 showed a significantly higher external void percentage (17.59 ± 1.76%) compared to Group 1 (9.05 ± 1.98%; *p* < 0.001), attributed to the stamp technique’s precise replication of occlusal micromorphology, misinterpreted as porosity by analysis software. Fractal dimension analysis revealed that the stamp technique resulted in the formation of a more complex structure. Restoration volumes (Group 1: 34.10 ± 8.09 mm^3^; Group 2: 35.52 ± 4.80 mm^3^; *p* = 0.639) and volumetric changes (Group 1: 5.91 ± 2.72 mm^3^; Group 2: 4.64 ± 1.31 mm^3^; *p* = 0.199) showed no significant differences. in conclusion, the stamp technique produced internal void percentages comparable to the conventional bulk-fill method in Class I restorations. The significantly higher external void percentage in the stamp technique group was due to the accurate replication of occlusal micromorphology, which was detected as porosity by analysis software. No significant differences were observed in volumetric changes of the occlusal surface before and after restoration between the two techniques, supporting the clinical viability of the stamp technique for precise occlusal restorations.

## 1. Introduction

Dental caries, a multifactorial disease, causes demineralization and destruction of hard dental tissues [1]. The primary goal of restorative dentistry is to replace lost tooth structure while restoring optimal form and function [2]. However, achieving this goal with traditional incremental obturation techniques can be challenging due to limitations in clinicians’ knowledge and technical skills [3,4].

The bulk-fill restorative technique refers to the placement of resin-based composite material in a single increment (typically up to 4–5 mm) to fill a cavity, as opposed to the traditional layering technique, which involves placing and curing the composite in multiple 2 mm increments [5]. Bulk-fill materials are specifically formulated to allow for adequate depth of cure, reduced polymerization shrinkage, and improved handling properties, enabling efficient restoration of posterior teeth [6]. This approach simplifies the restorative procedure, reducing the risk of errors associated with incremental placement, such as voids or contamination between layers [5,6,7]. Some studies report less marginal staining and fewer voids with bulk-fill techniques, likely due to fewer interfaces and a lower risk of incorporating air between increments [8,9].

Occlusal stability is critical for successful dental restorations, ensuring proper occlusion, harmonious interactions with adjacent teeth, and neuromuscular balance for comfortable chewing [10]. The stamp technique addresses occlusal challenges in Class I carious lesions. This method facilitates faster, more predictable restoration of the occlusal surface’s micromorphology, closely replicating natural tooth anatomy and minimizing discrepancies [10].

The stamp technique involves creating a pre-occlusal matrix prior to caries removal to capture the tooth’s original occlusal anatomy. This matrix is pressed onto the final composite layer before light curing [11]. It ensures precise replication with minimal risk of micro-gaps or voids and requires limited finishing procedures, such as polishing or occlusal adjustments [4,12]. This technique reduces operative time, which is an important consideration in pediatric dentistry for both patients and clinicians [13]. Unlike traditional direct composite fillings, which exhibit high polymerization shrinkage and demand advanced operator skills to achieve optimal occlusal harmony, the stamp technique minimizes these challenges [4]. It eliminates the oxygen-inhibited layer during polymerization, improving final restoration quality [4,11,12].

Microtomography is an effective and precise method for evaluating restoration quality by accurate measurement of void formation, eliminating the need for destructive sample evaluation [14].

Several methods have been employed to evaluate internal and external voids in bulk-fill composite restorations, each offering distinct advantages and limitations. Micro-computed tomography (micro-CT) is a non-destructive, high-resolution imaging technique. It enables precise three-dimensional quantification of void volume and distribution within restorations, making it ideal for assessing internal adaptation without damaging samples [15]. Scanning electron microscopy (SEM) provides detailed surface topography and can confirm the presence of external voids or microfeatures, though it requires destructive sample preparation and is limited to two-dimensional analysis [16]. Other techniques, such as optical coherence tomography (OCT), offer high-resolution imaging of interfacial gaps but are less effective for quantifying internal voids in deeper restorations [17,18]. OCT is less sensitive to radiopaque materials than micro-CT, but it is suitable for in vivo applications and can detect interfacial gaps and voids, especially at the adhesive interface. However, its penetration depth and resolution are lower than micro-CT, and it may underestimate void volume compared to micro-CT.

Micro-CT was selected for this study due to its ability to provide comprehensive, non-invasive volumetric analysis, allowing accurate measurement of both internal and external voids in bulk-fill restorations. However, comprehensive studies examining the bulk-fill stamp technique, particularly focusing on void formation and volumetric changes using micro-CT, remain limited. A deeper understanding of the stamp technique’s performance in these aspects will inform its clinical application for restoring occlusal caries lesions in pediatric patients.

There is a lack of studies examining the bulk-fill stamp technique as a restorative method, particularly focusing on the formation of internal and external voids and the volumetric changes in the occlusal surface after restoration. Gaining a better understanding of the advantages and disadvantages of the stamp technique will help clinicians make informed decisions regarding the best resin composite filling technique in pediatric patients with occlusal caries lesions.

The null hypothesis of the study was that the stamp technique, compared to the conventional bulk-fill technique, would result in similar internal and external void formation and restoration volumes due to the use of identical bulk-fill composite materials. No significant differences in volumetric changes on the occlusal surface before and after restoration would be expected between the two techniques.

The aims of the present study are:

1. Quantitative evaluation of the volume of external and internal voids formed during bulk-fill restorations using the stamp technique;
2. Assessment of the volumetric changes on the occlusal surface before and after restoration with the stamp technique.

## 2. Materials and Methods

### 2.1. Sample Size Calculation

The sample size was determined using a power calculation (G-Power statistical power analysis software, version 3.1.9.7) and based on previous studies [17]. Assuming a large effect size (Cohen’s d = 0.92) for differences in void percentages between groups, a power of 70% (β = 0.3), and a significance level of α = 0.05 (two-tailed), a minimum of 12 samples per group (n = 12 for Group 1: conventional bulk fill; n = 12 for Group 2: stamp technique) was calculated to detect significant differences using an independent samples *t*-test. This sample size was deemed sufficient to achieve adequate statistical power while accounting for the controlled in vitro conditions of the study.

### 2.2. Sample Selection

Twenty-four caries-free permanent molars were selected for the study, all extracted for orthodontic reasons within 6 months prior to the study (November 2024–April 2025). The samples comprised 14 maxillary (58.3%) and 10 mandibular (41.7%) permanent molars, randomly allocated to Group 1 (conventional bulk fill: 7 maxillary, 5 mandibular) and Group 2 (stamp technique: 7 maxillary, 5 mandibular). After extraction, teeth were cleaned with ultrasonic scaling and pumice, then stored in 0.1% thymol solution at 4 °C to prevent bacterial growth and maintain tissue integrity, with storage duration not exceeding 6 months to ensure structural stability. After the experiment, the teeth were disposed of in accordance with biohazard waste protocols. The study procedures were approved by the Medical University of Sofia KENIMUS Ethics Committee (Approval Number: No 1936/8 May 2025).

### 2.3. Micro-CT Scanning

Each sample was scanned before the restoration to establish a baseline tooth structure volume. The teeth were mounted in a custom jig to ensure consistent positioning during the scanning. The roots of the specimens were fixed using A-silicone impression material. The crowns were scanned with a SkyScan 1272 Desktop X-ray Microtomography (Bruker, Billerica, MA, USA) with a nominal resolution of 1448 pixels, an X-ray tube voltage of 95 kV, 360° rotation with 0.6° rotation step, and a 1 mm copper filter. A conical beam with a voxel size of 10 μm was used. At this resolution, the crowns were projected over their entire length into the detector field.

After scanning, a three-dimensional reconstruction of the tooth crown was performed by superimposing a series of two-dimensional images using the SkyScan NRecon software (version 2.2.0.6, Bruker, Kontich, Belgium) accelerated by a GPU. Ring artifact reduction of 4, beam hardening of 55%, and post-scan correction for slow geometry change were applied.

### 2.4. Cavity Preparation

Standardized occlusal cavity preparations (Class I, 4 mm depth, 5 mm mesiodistal width, 4 mm buccolingual width) using a high-speed handpiece with a diamond bur under water cooling were made.

Afterward, the teeth were randomly assigned to two groups:

- Group 1: Bulk-fill restorative technique
- Group 2: Stamp technique with bulk-fill composite.

### 2.5. Cavity Preparation, Etching, and Adhesive Protocol

Each cavity was etched with 37% phosphoric acid (3M^TM^ Scotchbond^TM^ Universal Adhesive, 3M ESPE, Athlone, Ireland) for 30 s on enamel and 15 s on dentin. The teeth were then rinsed with water for 30 s and gently air-dried. A layer of adhesive (Adhese Universal VivaPen, Ivoclar Vivadent, Schaan, Liechtenstein) was applied using a microapplicator and polymerized with a light-curing unit (Elipar^TM^ Freelight 2, 3M ESPE, Athlone, Ireland) at an intensity of 1000 mW/cm^2^ for 20 s.

### 2.6. Restoration Procedure

#### 2.6.1. Group 1: Bulk-Fill Composite Restoration

A composite restoration (Tetric EvoCeram^®^ Bulk Fill, Ivoclar Vivadent, Schaan, Liechtenstein) was placed in a single layer. The occlusal surface was manually contoured to replicate anatomical morphology and then light-cured for 20 s. An additional 10 s polymerization was performed using glycerin gel (Cercamed, Stalowa Wola, Poland) to eliminate the oxygen-inhibited layer. The restoration was finished and polished using abrasive discs of decreasing grit (Sof-Lex^TM^ Pop-On, 3M ESPE, St. Paul, MN, USA).

#### 2.6.2. Group 2: Stamp Technique with Bulk-Fill Composite

Pre-Restorative Stamp Creation: Prior to cavity preparation, a pre-restorative stamp of the occlusal surface was created using a liquid rubber dam (Opaldam, Ultradent Products Inc., South Jordan, UT, USA) to capture the original anatomical contours. After the cavity preparation and the adhesive protocol, a bulk-fill composite (Tetric EvoCeram^®^ Bulk Fill, Ivoclar Vivadent, Schaan, Liechtenstein) was placed in a single 4 mm layer, ensuring complete adaptation to the cavity walls. A thin layer of Teflon tape then covered the material and the occlusal surface. The premade stamp was pressed onto the uncured composite to recreate the occlusal anatomy. Any excess material was removed from the surface prior to polymerization. The composite was once light-cured for 10 s using the Elipar^TM^ Freelight 2 LED curing unit (3M ESPE, Athlone, Ireland) at 1000 mW/cm^2^ through the stamp and for another 10 s after the removal of the stamp and the Teflon paper (Supplementary Materials: Video S1). An additional 10 s polymerization was performed using glycerin gel. The restoration was finished and polished as described for Group 1.

The materials used in the study are presented in Table 1.

### 2.7. Post-Restoration Micro-CT Scanning

After the restoration of each tooth, a micro-CT scan was performed using the same position and parameters as the pre-restoration scan to maintain consistency in data collection. The quantitative assessment of the dental restorations was carried out using image analysis software (CtAn, Bruker, Kontich, Belgium, version 1.23.01), ensuring precise measurements of voids and volume changes in the tooth. Global thresholds were selected by visual matching with grayscale images. Fractal dimension was calculated in 3D by the Kolmogorov box counting method [19].

Voids, or air bubbles, were identified through grayscale thresholding, allowing for the differentiation between the low-density air and the denser composite and tooth structures. The datasets before and after restoration were binarized, aligned, and the resulting differences were stored as a separate dataset (Dataviewer, Bruker, Billerica, MA, USA), which was then analyzed and its volume measured with CTAn visualization software.

**Figure 1:**
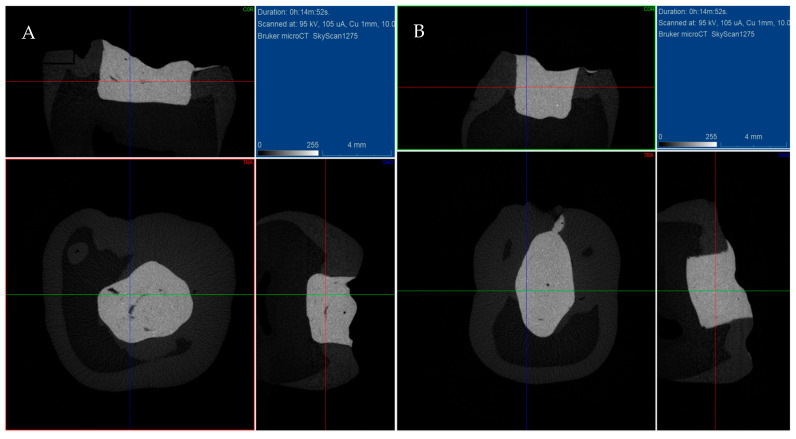
Microtomographic views of restored tooth crowns: (**A**) stamp technique; (**B**) conventional bulk-fill technique.

Figure 1 presents microtomographic images of the two groups.

### 2.8. Statistical Analysis

Statistical analysis was performed using IBM SPSS Statistics, Version 19.0 (IBM Corp., Armonk, NY, USA). The normality of data distribution was confirmed using the Shapiro–Wilk test (*p* > 0.05), and homogeneity of variances was verified with Levene’s test (*p* > 0.05). An independent samples *t*-test was used to compare the percentages of internal, external, and total voids, as well as the restoration volumes and volumetric changes between Group 1 (conventional bulk-fill technique) and Group 2 (stamp technique). To calculate the relationship between total voids formed and volumetric changes, the Pearson correlation coefficient was calculated. The significance level (α) was set at 0.05 for all comparisons, with *p*-values below 0.05 considered statistically significant.

## 3. Results

**Figure 2:**
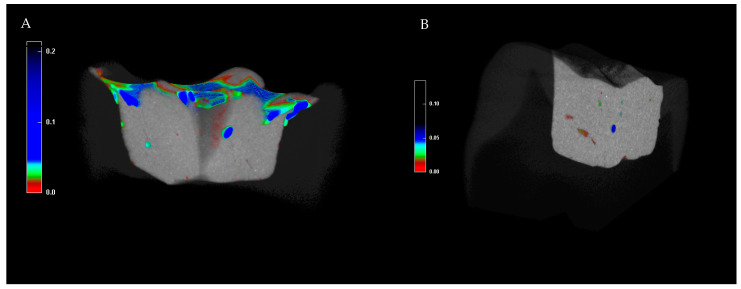
Representative micro-CT images of restoration with stamp (**A**) and conventional (**B**) bulk-fill technique with superimposed voids in different color, depending on their size.

Figure 2 shows the micro-CT images of the studied groups, with voids of varying sizes indicated by different colors.

Table 2 presents the percentage of internal, external, and total voids in the two studied groups.

The mean internal void percentage in Group 1 (0.38 ± 0.22%) is slightly lower than in Group 2 (0.39 ± 0.30%; *p* = 0.914), with no statistically significant difference, indicating comparable performance of both techniques in minimizing internal voids. However, Group 2 (stamp technique) shows a significantly higher mean external void percentage (17.59 ± 1.76%) compared to Group 1 (9.05 ± 1.98%; *p* < 0.001), with the mean total void percentage reflecting a similar pattern. The mean total void percentage followed a similar pattern, with Group 2 (17.91 ± 1.68%) significantly higher than Group 1 (9.39 ± 2.07%; *p* < 0.001).

**Figure 3:**
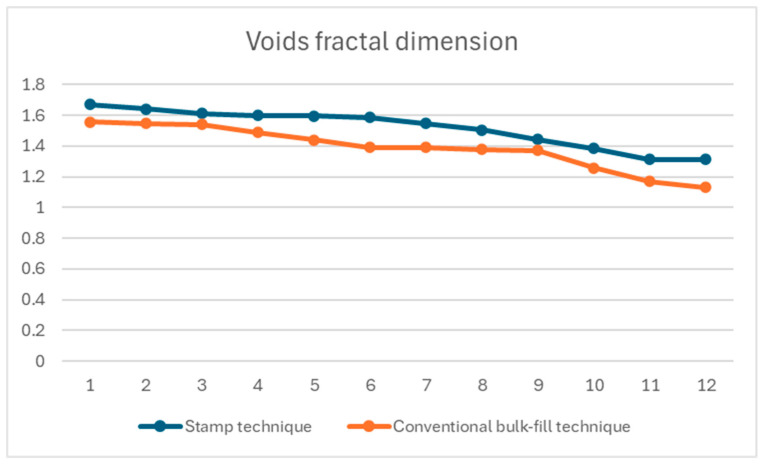
Fractal analysis of the restoration voids in stamp and conventional bulk-fill techniques.

Figure 3 shows the fractal dimension analysis of voids for both the stamp and conventional bulk-fill techniques.

The mean fractal dimension was 1.52 ± 0.13 for the stamp technique and 1.39 ± 0.14 for the conventional bulk-fill technique. Statistical analysis revealed a significant difference between the two groups (*p* = 0.03), with the stamp technique resulting in a more complex structure.

Table 3 describes the volume of fillings in the two study groups and the differences in the volumes of the dental crowns before and after the fillings were made.

The two groups indicate no statistically significant difference in restoration volume, suggesting comparable filling volumes. The total volume of the teeth before and after the placement of the bulk-fill restoration also shows no significant differences. The SD values of Group 2 are lower, which suggests greater consistency in the stamp technique, potentially due to its precision in replicating occlusal anatomy, though the differences in means are not statistically significant. The Pearson correlation showed a positive link between restoration volume differences and total voids with the stamp technique (r(10) = 0.71, *p* = 0.02), but not with the conventional bulk-fill method (r(10) = −0.60, *p* = 0.07).

**Figure 4:**
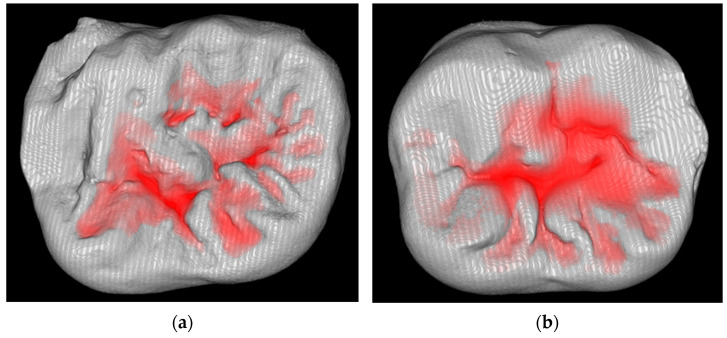
Differences between original occlusal anatomy and final restoration. (**a**) Stamp technique bulk-fill restoration. (**b**) Conventional bulk-fill restoration.

Figure 4 represents the restoration on the occlusal surfaces with superimposed differences from the original occlusal anatomy.

## 4. Discussion

The advantages of the stamp technique in the restoration of Class I cavities include highly accurate reproduction of the original occlusal anatomy, which can improve both esthetics and functional outcomes [14]. Clinical case series and follow-up studies have shown that stamp technique restorations exhibit good longevity, wear resistance, minimal marginal discrepancies, and sustained occlusal morphology over several years [20]. Additionally, the use of stamps has been shown to minimize the removal of healthy tooth structure during finishing, as the stamp is more dimensionally stable and allows for precise adaptation of the composite [21].

The present study aimed to quantitatively evaluate the volume of internal and external voids formed during bulk-fill obturation using the stamp technique compared to the conventional bulk-fill technique and assess volumetric changes in tooth structure before and after restoration. The stamp restoration technique results in more external voids on the occlusal surface than the other group, due to its superior ability to replicate the intricate details of the occlusal tooth’s natural anatomy. This technique meticulously captures the occlusal anatomy, enhancing the replication of natural tooth structure. These microscopic features, inherent to the tooth’s structure, are faithfully reproduced during restoration. When analyzed by evaluation software, these replicated details are often interpreted as “voids” due to their fine-scale and irregular texture. In contrast, other restoration techniques may smooth over or fail to capture these minute irregularities, resulting in fewer detected voids but a less accurate reproduction of the tooth’s natural surface. The stamp technique’s precision in restoring these microfeatures enhances its effectiveness, ensuring a restoration that closely mimics the original tooth structure, which is critical for both functional and esthetic outcomes.

No significant difference was observed in the percentage of internal voids between the groups. This finding suggests that both the conventional bulk-fill and stamp techniques achieve comparable internal adaptation to cavity walls. The similar internal void percentages are likely due to both groups using the same bulk-fill composite and adhesive protocols, which reduce polymerization shrinkage and ensure good material flow into the cavity [22]. The results are consistent with previous studies, which report that bulk-fill composites, with their enhanced depth of cure and reduced shrinkage stress, effectively minimize internal voids compared to incremental layering techniques [23].

Void formation in bulk-fill dental restorations is a well-characterized phenomenon that can influence stress distribution and failure modes. Micro-CT and optical coherence tomography studies demonstrate that voids—whether internal or external—are present in both bulk-fill and incremental techniques, but their incidence and volume are generally lower in bulk-fill restorations, especially when flowable materials are used or heat is applied during placement [15,18]. The presence of voids acts as stress concentrators within the restoration, leading to localized increases in tensile and shear stresses under occlusal loading, which can predispose the restoration to crack initiation and propagation, marginal breakdown, and ultimately restoration failure [24,25].

Finite element analyses confirm that voids disrupt the uniform distribution of occlusal forces, resulting in higher stress gradients at the restoration–tooth interface and within the restorative material itself [26]. This altered stress distribution can compromise occlusal accuracy, as micro-movements and deformation around voids may lead to premature wear, marginal gaps, and loss of anatomical contour. Failure modes associated with voids include cohesive fractures within the composite, adhesive failures at the interface, and mixed failures involving both tooth and restoration [27,28].

The restoration volumes were found to be similar between the samples (Table 3). This indicates that both techniques achieve comparable filling volumes, adhering to the standardized cavity preparation and utilizing the same bulk-fill composite. Similarly, the volumetric change in the crown before and after restoration showed no significant difference. These findings suggest that both techniques effectively restore the lost tooth structure without substantial volumetric discrepancies. The stamp technique excels in replicating occlusal micromorphology with high accuracy, offering significant advantages in achieving occlusal stability and harmonious interactions with adjacent teeth [11,12].

The restoration of the occlusal anatomy depends fundamentally on the clinician’s skills, working conditions, and knowledge of restorative techniques [29,30]. Achieving accurate occlusal morphology requires a thorough understanding of occlusal principles, anatomy, and function, as well as proficiency in the chosen restorative method [29,30]. The clinician’s expertise is critical, encompassing technical proficiency, attention to detail, and familiarity with the restorative technique, such as the stamp or conventional bulk-fill method. Skilled clinicians can better manipulate materials and tools to precisely replicate the intricate details of the occlusal surface, including porosities, microroughness, and anatomical contours. Clinicians should use the stamp technique carefully with deep occlusal fissures, as these can cause incomplete composite infusion and gaps. Proper cavity preparation and stamp design help ensure good material flow in complex cases.

Limitations: This study was conducted under controlled in vitro conditions, which do not fully replicate the clinical environment. Factors such as patient movement, saliva contamination, time constraints, and limited visibility in vivo may impact the performance of both the stamp and conventional bulk-fill techniques, potentially altering the outcomes observed in this study.

The significantly higher percentage of external voids in the stamp technique group suggests that this methodology may not be suitable for assessing voids in this application, as the results reflect occlusal microfeatures rather than actual voids. Furthermore, while the study highlights the importance of clinician expertise, it does not address potential variability in operator skill or experience. Differences in technical proficiency among clinicians could influence the quality of restorations and the consistency of results in clinical settings.

While the increased external void percentage in the stamp technique group is attributed to the software’s misinterpretation of occlusal groove morphology, future studies using scanning electron microscopy (SEM) could validate this hypothesis by providing detailed surface analysis to distinguish between true voids and replicated natural microfeatures.

Furthermore, the absence of thermal cycling and mechanical fatigue testing is a limitation, as these tests could evaluate long-term void stability and restoration durability under simulated oral conditions. Due to limited funding, these experiments were not feasible, but they represent critical avenues for future studies to assess the performance of the stamp and conventional bulk-fill techniques.

Additionally, the lack of scanning electron microscopy and micro-tensile bond strength testing limits the interfacial analysis. These tests, which could provide detailed visualization and quantification of bonding integrity, were not feasible due to funding constraints. Future studies should incorporate SEM and μTBS testing to validate the interfacial characteristics of the stamp and conventional bulk-fill techniques.

## 5. Conclusions

The stamp technique produced internal void percentages comparable to the conventional bulk-fill method in Class I restorations. The significantly higher external void percentage in the stamp technique group was due to the accurate replication of occlusal micromorphology, which was detected as porosity by analysis software. No significant differences were observed in volumetric changes of the occlusal surface before and after restoration between the two techniques, supporting the clinical viability of the stamp technique for precise occlusal restorations.

## Figures and Tables

**Table 1:** Materials used in the study and their composition.

Material	Composition	Manufacturer
Tetric EvoCeram^®^ Bulk Fill	Bis-GMA, UDMA, barium glass, ytterbium trifluoride, mixed oxide, and prepolymerized fillers, photoinitiator	Ivoclar Vivadent, Schaan, Liechtenstein
3M^TM^ Scotchbond^TM^ Universal Etchant	37% phosphoric acid gel	3M ESPE, Athlone, Ireland
Adhese Universal VivaPen	10-methacryloyloxydecyl dihydrogen phosphate (10-MDP) as the functional monomer, 2-hydroxyethylmethacrylate (HEMA), other methacrylate monomers, water, ethanol, and initiators	Ivoclar Vivadent, Schaan, Liechtenstein
Glycerin Gel	Glycerin, water	Cercamed, Stalowa Wola, Poland
Sof-Lex^TM^ Pop-On Discs	Aluminum oxide abrasive particles, flexible backing	3M ESPE, St. Paul, MN, USA

**Table 2:** Void percentages in bulk-fill restorations.

Groups	Internal Voids (%) Mean ± SD	External Voids (%) Mean ± SD	Total Voids (%) Mean ± SD
Group 1—conventional bulk-fill technique	0.38 ± 0.22	9.05 ± 1.98	9.39 ± 2.07
Group 2—stamp technique	0.39 ± 0.30	17.59 ± 1.76	17.91 ± 1.68
*t*-test	*p*_1,2_ = 0.914	*p*_1,2_ < 0.001	*p*_1,2_ < 0.001

**Table 3:** Restoration volumes and volumetric changes.

Groups	Restoration Volume Mean ± SD	Difference Volume Mean ± SD
Group 1—conventional bulk-fill technique	34.10 ± 8.09	5.91 ± 2.72
Group 2—stamp technique	35.52 ± 4.80	4.64 ± 1.32
*t*-test	*p*_1,2_ = 0.639	*p*_1,2_ = 0.199

## Data Availability

The original contributions presented in this study are included in this article/Supplementary Materials, and further inquiries can be directed to the corresponding author.

## Supplementary Materials

The following supporting information can be downloaded at: https://www.mdpi.com/article/10.3390/ma18174027/s1, Video S1: Stamp restoration technique.

## Abbreviations

The following abbreviations are used in this manuscript:

Micro-CT	Micro-computed tomography
OCT	Optical coherence tomography
SEM	Scanning electron microscopy
